# Structural stigma and its impact on healthcare for consumers with borderline personality disorder: protocol for a scoping review

**DOI:** 10.1186/s13643-021-01580-1

**Published:** 2021-01-11

**Authors:** Pauline Klein, Alicia Kate Fairweather, Sharon Lawn, Helen Margaret Stallman, Paul Cammell

**Affiliations:** 1 Discipline of Behavioural Health, College of Medicine and Public Health, Flinders University, Adelaide, South Australia 5001 Australia; 2 Thompson Insititute, University of the Sunshine Coast, 12 Innovation Pkwy, Birtinya, Queensland 4575 Australia; 3 The Royal Melbourne Hospital, Parkville, Victoria 3050 Australia

**Keywords:** Borderline personality disorder, Stigma, Health systems, Healthcare services, Crisis care, Clinicians

## Abstract

**Background:**

Structural stigma in health systems experienced by consumers diagnosed with Borderline Personality Disorder (BPD) is a widespread phenomenon that causes major health inequities and harm for this population. Structural stigma in this context relates to institutional policies, cultural norms, and organizational practices that limit consumers’ access to health services, quality of care, and capacity to achieve optimal health and well-being. BPD is a serious mental illness with high morbidity and mortality, characterized by instability in interpersonal relationships, self-image, and emotional and behavioral deregulation, which stem from significant traumatic childhood/life events, and/or biological etiologies. The objectives of this scoping review are to explore the international literature on structural stigma in healthcare systems specific to BPD, and to provide an overview of the impact of structural stigma on health services for BPD consumers and their carers/families.

**Methods:**

This scoping review will follow the Joanna Briggs Institute (JBI) scoping review guidelines. We will search the following electronic databases (from inception onwards): MEDLINE, CINAHL, PsycINFO, Scopus, Cochrane Library, and JBI-Evidence databases. Grey literature will be identified through the Google search engine. We will include all types of literature in English, published and unpublished, including any study design, reviews, clinical practice guidelines, policy reports, and other documents. No restrictions on publication date of sources of evidence will be applied. International literature should examine structural stigma associated with BPD in any healthcare setting such as, outpatients, inpatients, primary health care, or community-based facilities. Two reviewers will independently screen all titles, abstracts, and full-text citations. Quality appraisal of the included sources of evidence will be assessed using the Mixed Methods Appraisal Tool (MMAT) 2018 version. Data analysis will involve quantitative (e.g., frequencies) and qualitative (e.g., thematic analysis) methods.

**Discussion:**

This review is anticipated to enhance both identification and understanding of those structures in health systems (i.e., institutional policies, cultural norms, and practices) that manifest and perpetuate stigma experienced by consumers with BPD and their carers/families. The findings can be used to inform future research, policy, and practice relating to stigma reduction strategies that can be adopted to improve the provision of BPD-responsive services and care for this population.

**Systematic review registration:**

Open Science Framework (https://osf.io/bhpg4).

**Supplementary Information:**

The online version contains supplementary material available at 10.1186/s13643-021-01580-1.

## Background

A dominant stigma discourse, perpetuated within healthcare systems towards people with mental illness [1], continues to undermine the lives of consumers and their carers/families worldwide [2]. Structural stigma in healthcare systems are the “societal-level conditions, cultural norms, and institutional policies that constrain the opportunities, resources, and wellbeing of the stigmatized” ([3] p742). Stigma occurs and is normalized within society [4] through social relationships and interactions [1], including organizations and institutions that generate and perpetuate notions of difference. This includes persons deemed to be “different from others” (sic), in a manner that is widely discrediting [1]. For example, normative structures within healthcare systems develop and shape expectations regarding what is “accepted” behaviour within that context. It is these expectations that historically created marked differences between people with mental illness and the general population; consequently manifesting structural stigma towards people with mental illness [5].

Extant at the macro-social level [2, 3, 6], stigma perpetuates deep-seated stereotypes, prejudice, and discriminatory influences on institutions’ organizational policy, culture, and practice [2, 4]. This includes, for example, clinical policies and practices that contribute to the stigmatization of consumers with mental illness [7, 8]. Policy decisions play a significant role in limiting the availability of services for people with mental illness, which is evident in the inequitable distribution of resources allocated to mental health services relative to general medical services [2]; analogously, less funding is dedicated to research and treatment of BPD, compared with other mental illnesses [9].

Consumers with a diagnosis of BPD are particularly vulnerable to the effect of structural stigma and, indeed, report experiencing greater levels of stigma within health systems compared to their counterparts with other mental illnesses [7, 10]. BPD is a serious mental illness typically characterized by significant traumatic childhood/life events and/or biological etiology serving to catalyze a pattern of instability in domains of interpersonal relationships, self-image, and emotional and behavioral (de)regulation [11]. The global estimates of the prevalence of BPD range from 1 to 5.9% in the general population [12–15], 10% of the patients in outpatient settings, and 15–22% of the patients in inpatients settings [12, 16, 17]. People with a diagnosis of BPD experience overwhelming distress [18] and also represent a high risk group for suicidality (i.e., suicidal thoughts, self-harm, suicide attempts), with approximately 10% of people with BPD dying by suicide [19]. Recurrent suicidality is often reported to be the reason why people with BPD and their carers/families frequently access healthcare services [20]. Help-seeking, including frequent presentations to emergency departments in response to suicidality, is common among consumers with BPD [20]. Although help-seeking has been identified as a healthy coping strategy for assisting consumers with BPD to cope with overwhelming distress [18], current treatment and care responses are often inadequate in meeting the specific needs of this population [20, 21]. In addition, help-seeking is commonly met by stigma-related experiences in relation to the BPD diagnosis [20]. Findings from a national survey of consumers with BPD and their carers/families identified that currently BPD-related health services are limited and constitute poor quality care, especially during times of crisis [22, 23]. In addition, over 50% of carers reported instances where the person they care for attempted to seek help for suicidality, but were refused hospitalization by some health providers [23]. These discriminatory experiences exacerbate and re-traumatize consumers’ distress [15], reinforcing consumers’ intense feelings of anxiety and hopelessness [24] and perpetuating the cycle of problems carers/families and clinicians seek to manage or treat [25].

Studies examining clinicians’ attitudes towards consumers with BPD highlighted the challenges experienced by clinicians working with this population [7, 26], particularly BPD consumers who present to health services in crisis [26]. A study assessing mental health staff attitudes towards consumers with BPD found that over 80% of staff viewed this population as difficult to work with, and indeed, more difficult to treat than consumers with other mental illnesses [10]. Clinicians disclosed experiencing strong negative emotions towards consumers with BPD, such as feelings of frustration, anger [27], and powerlessness [26]. In addition, some clinicians have described consumers with BPD as manipulative—that is, clinicians believe people with BPD have more control of their emotions and behaviors than consumers with other mental illnesses; and they misbehave—rather than their behavior being an expression of mental illness [28, 29]. Moreover, there are reports of some clinicians denying people with BPD treatment [15, 30]. These findings support recommendations for BPD-specific education and training to enhance clinicians’ knowledge and practice in relation to their treatment approach to working with this population [15]. Notably, research shows that clinicians *are* interested in attending training to help them to more effectively treat consumers with BPD [7, 8, 10].

Overall, stigma associated with restricted access to health services [2, 6, 31] and suboptimal quality of care [15, 29] undermine the diagnosis, treatment, and health outcomes of BPD consumers [32]. A recent systematic review exploring stigma at the interface of mental health care identified several fundamental processes contributing to the creation and perpetuation of stigma towards people with BPD. These included “stigma related to diagnosis and disclosure, perceived untreatability, and demand for services; stigma as a response to feeling powerless; stigma due to preconceptions of the BPD patient; and low BPD health literacy” [26] (p4-16). Recommendations for addressing these complex health inequities and overcoming stigma in mental health settings involved the need to enhance empathy among clinicians through the development of a conceptual framework for understanding the complexities of BPD-based behaviors, and the resultant stigma, within healthcare interactions among consumers with BPD, their carers/families, and clinicians [26].

The decision to conduct a scoping review was based on the premise that while there is a diverse range of literature available on the lived experiences of BPD-related stigma in health settings among BPD consumers, their carers/families, and clinicians; there is limited knowledge on the mechanisms that influence structural stigma in healthcare systems, and the impact that these processes have on the delivery of quality services to consumers with BPD and their carers/families. Conducting this scoping review will allow us to broadly map the extent of the evidence relating to BPD-related structural stigma in healthcare systems and identify solutions to address the systemic policies, procedures, and practices that contribute to stigma-related outcomes.

### Study aims

This scoping review aims to enhance identification and understanding of the structures in healthcare systems, including the institutional policies, cultural norms, and pervasive organizational practices that contribute to stigma [3, 6] experienced by consumers with BPD and their carers/families. Our objectives are to explore the international literature on structural stigma specifically in relation to BPD in healthcare systems by providing an overview of the current evidence. This will involve assessing how structural stigma impacts the provision of health services and quality of care for consumers with BPD, their carers/families, and clinicians.

The primary research question is, how does structural stigma associated with a diagnosis of BPD impact on access to health services and quality of care for people with BPD, their carers/families, and clinicians? Secondary research questions will be explored to facilitate a deeper understanding of the key factors, barriers, and mechanisms contributing to the pervasive stigma in healthcare systems experienced by this population, as follows: (1) What are the perspectives and experiences of structural stigma among consumers with BPD, their carers/families, and clinicians? (2) What are the specific drivers and facilitators influencing the manifestation and perpetuation of structural stigma in healthcare systems towards consumers diagnosed with BPD, and the implications for research, policy, and practice?

## Methods

This review protocol was registered within the Open Science Framework (registration ID: https://osf.io/bhpg4) and is being reported in accordance with the reporting guidance provided in the Preferred Reporting Items for Systematic Reviews and Meta-Analyses Protocols (PRISMA-P) statement [33] (see checklist in Additional file 1). The scoping review methodology will be conducted in accordance with the JBI guidelines for scoping reviews [34] and Arksey and O’Malley’s [35] five-step process for scoping reviews.

### Eligibility criteria

The Population-Concept-Context (PCC) framework [34] will be used to align the study selection with the research question. To be included in the review, sources of evidence must include citations that focus on BPD-related stigma in healthcare systems. This includes studies examining the perspectives and lived experiences of BPD consumers, carers/families, and/or clinicians who treat consumers with BPD in healthcare settings; consumers aged 12 years or older who have a diagnosis of BPD and carers/families of people with BPD; and clinicians working in clinical outpatients, inpatients, or community-based services. The phenomenon of interest for this scoping review is the concept of structural stigma—this will subsume stigma maintained by some clinicians in relation to consumers with BPD and their carers/families within the context of crisis care. The rationale underpinning the decision to include studies with a focus on stigma as an outcome is to enable the current review to identify and map the key outcomes of structural stigma relevant to a diagnosis of BPD in healthcare systems. Included studies will be BPD-specific and focus on the impact of stigma on access to health services and quality of care for consumers with BPD and their carers/families; the identification of contributing factors associated with structural stigma in healthcare systems; and policy and funding allocation associated with BPD-related services in healthcare settings. In addition, studies that examine structural stigma associated with BPD in the context of healthcare settings will be included such as, outpatients, inpatients, primary health care, or community-based facilities.

Sources of evidence will also be included if they meet the following criteria:

#### Inclusion criteria

- Written in English language only
- International peer-reviewed journal articles (including mixed-methods, quantitative and qualitative study designs) and reviews
- Non peer-reviewed literature including clinical practice guidelines and other government reports relating to the treatment of consumers with BPD in healthcare services
- There are no restrictions to the publication dates of citations.

Sources of evidence that will be excluded if they do not meet the following criteria:

#### Exclusion criteria

- Editorials, commentaries, books, theses, conference abstracts, abstracts without full-text available, and presentations
- Studies with a focus on mental illnesses that do not include BPD
- Studies on brain abnormalities relating to BPD
- Studies on structural stigma in non-clinical settings
- Studies on self-stigma relating to BPD and other mental illnesses.

The rationale for these decisions was our focus on identifying existing empirical evidence on structural stigma in healthcare systems, and its impact on healthcare specific to consumers with BPD and their carers/families.

### Information sources and search strategy

The primary source of literature will be a structured search of electronic databases (from inception onwards): MEDLINE (Ovid), CINAHL (EBSCO Connect), PsycINFO (Ovid), Scopus (Elsevier), Cochrane Library (Wiley), and JBI Evidence-Based Database (Ovid). The secondary source of potentially relevant material will be a search of the grey or difficult to locate literature, including Google search engine, to identify other relevant citations such as clinical practice guidelines and other government reports (Additional file 2). The references of included citations will be hand-searched to identify any additional evidence sources. The search strategy was designed by a research librarian and peer-reviewed by using the Peer Review of Electronic Search Strategies (PRESS) checklist [36]. Draft searches were executed in PsycINFO to test the search text word terms and subject heading combinations. The search strategy was refined following analysis of relevant citation key words, resulting in a comprehensive search strategy to identify all existing peer-reviewed articles relating to the topic of interest. The search was further tested to ensure that at least 10% of the citations were relevant to the topic and the 10 test articles were located in the final search. A draft search strategy for PsycINFO (Ovid) is provided in Additional file 3.

### Selection of sources of evidence

All citations identified from the search will be collated and uploaded into *EndNote* v.9. Citations will be uploaded into *Covidence* and deduplicated by the lead author (PK). Two independent reviewers (PK and AKF) will screen the title, abstracts, and full-text citations against the defined selection criteria to identify relevant studies. Full-text citations of selected studies will be retrieved via *Covidence* and assessed against the inclusion criteria. Full-text citations that are deemed ineligible will be omitted in accordance with the exclusion criteria and reported in the final scoping review paper. Any discrepancies in decisions regarding the inclusion of studies will be accessed and resolved by a third reviewer (SL) with clinical expertise in the field of the mental health. Results of the search will be presented in a PRISMA Flow Diagram generated by *Covidence* and reported in full in the final scoping review paper.

### Critical appraisal

Quality appraisal of studies will be conducted to support the purpose of this scoping review, which is to examine existing and complex structural issues that impact healthcare services for consumers with BPD and to establish the methodological quality and validity of the included studies. MMAT v.2018 will be used to conduct the quality appraisal [36]. MMAT is a checklist developed to appraise the methodological quality of empirical studies including, quantitative, qualitative, and mixed methods studies for inclusion in systematic mixed study reviews [36]. One reviewer (PK) will conduct the quality appraisals, and a second reviewer (AKF) will review and highlight any discrepancies. If there are any discrepancies concerning quality assessment decisions and the two investigators performing the appraisals cannot reach a consensus, the discrepancies will be resolved through discussion and consultation with a third reviewer (SL).

### Data extraction

Data extraction tools will be used to chart extracted data from the included studies. A standard data extraction tool will be used to capture data including, author, year, country; population type and sample size; clinical area and health setting; study design; data collection and methods; and main findings (Additional file 4). A second data extraction tool will be used to capture relevant data specific to BPD-related structural stigma in healthcare systems including any systemic policies, procedures, practices, and funding affecting healthcare services for consumers with BPD and their carers/families (see Additional file 5). These data extraction tools will be piloted on a small number of selected studies. Piloting of these initial studies will be charted by two reviewers independently (PK and AKF). The data extraction tools and processes will then be reviewed and updated according to any lessons learned during the piloting process. Similar to the critical appraisal process, one reviewer (PK) will complete data extraction by populating data from the rest of the included studies, and the second reviewer (AKF) will review, highlight, and discuss any discrepancies to be resolved with the reviewers as required. No processes for obtaining data from authors will be conducted in this scoping review.

### Data synthesis

The results of the scoping review will be synthesized into a narrative summary relating to the study objectives, research questions, and eligibility criteria (PCC). Data analysis will involve quantitative data being summarized using descriptive statistics and frequencies [37], and thematic analysis of qualitative data to organise, categorize, and interpret the key themes and patterns emerging from the data [38]. We will use tables and mind maps to summarize key article findings, their characteristics, definitions of BPD-related structural stigma, associated factors, and other main findings based on the data extraction criteria. Suggestions for future research based on the study findings will also be summarized. Implications for future research, policy, and practice in addressing BPD-related structural stigma to improve health services for consumers with BPD and their carers/families will also be summarized.

## Discussion

The purpose of this scoping review is to map existing international literature on structural stigma in healthcare systems specific to BPD and synthesize the findings on the impact of stigma on the delivery of responsive healthcare services for consumers with BPD and their carers/families. Evidence has indicated that institutionalized stigma affects consumers with BPD—particularly those where a crisis results in their presentation to health services, such as emergency departments—more than consumers with other mental illnesses [4, 7, 22, 24]. By completing this review, we hope to gain a better understanding of consumers, their carers/families, and clinicians lived experiences of stigma, and the mechanisms that perpetuate stigma in healthcare systems. Understanding the lived experiences of consumers with BPD and their carers/families can help to identify the specific needs of this population as well as the current gaps in health services [22, 24]. Exploring clinicians lived experiences of working with consumers diagnosed with BPD will also be beneficial in broadening our understanding of the specific challenges, barriers, and gaps in knowledge and skills experienced by various healthcare providers [8, 10]. This can assist in developing recommendations for BPD-related education and training for clinicians to enhance the delivery of effective services that meet the needs of consumers with BPD and their carers/families [26]. Triangulating perspectives and lived experiences of stigma among consumers, carers/families, and clinicians has been identified as a relatively new approach to conceptualize the complex mechanisms that impact on clinical practice in healthcare systems [39]. Further, this review will inform future research, policy, and practice relating to stigma reduction strategies that can be adopted to improve quality person-centered care and consumer outcomes [18]. As this scoping review is one of two intended reviews designed to examine BPD-related stigma in healthcare systems, these findings will also inform a systematic review on the impact of anti-stigma interventions in addressing BPD-related prejudice in healthcare settings [28].

Potential limitations in this review may involve the exclusion of full-text publications that are unable to be accessed free of charge, and older dated publications may be missed. No anticipated operational issues in relation to the completion of scoping review processes are expected. The scoping review methodology will enable us to make relevant amendments (and document those changes) to the scoping protocol as required.

Findings from this scoping review will make a valuable contribution towards recommendations for addressing BPD-related stigma in healthcare systems to improve the delivery of person-centered care and health outcomes for this population [8, 40]. Findings from this review will be disseminated through publications in peer-reviewed journals, conferences, and by partner organizations. The proposed scoping review will be reported in accordance with the reporting guidance provided in the PRISMA extension statement for scoping reviews (PRISMA-ScR) [41]. Any amendments made to this protocol when conducting the review will be outlined and reported in the Open Science Framework and in the final manuscript.

## Supplementary Information

**Additional file 1.** PRISMA-P 2015 Checklist.

**Additional file 2.** Grey literature search strategy.

**Additional file 3.** Draft search strategy for PsycINFO database.

**Additional file 4.** Draft data extraction tool of included studies.

**Additional file 5.** Draft data extraction tool for structural issues associated with BPD-related stigma in healthcare systems.

## Data Availability

Not applicable.

## Abbreviations

BPD: Borderline personality disorder
CINAHL: Cumulative Index to Nursing and Allied Health Literature
JBI: Joanna Briggs Institute
MMAT: Mixed Methods Appraisal Tool 2018 version
PCC: Population-Concept-Context framework
PRESS: Peer Review of Electronic Search Strategies
PRISMA-ScR: Preferred Reporting Items for Systematic Reviews and Meta-Analyses; extension for Scoping Reviews: checklist and explanation
