# Supplementary material for: Structural stigma and its impact on healthcare for consumers with borderline personality disorder: protocol for a scoping review
Source: Syst Rev. 2021 Jan 11;10:23. doi: 10.1186/s13643-021-01580-1 (PMC7798332; doi:10.1186/s13643-021-01580-1)
Supplement: Supplementary file 2 — Additional file 2. Grey literature search strategy. [file 13643_2021_1580_MOESM2_ESM.docx]

**Additional file 2: Grey literature search strategy**

Key words:

“Borderline personality disorder” “BPD” “consumer” “carer” “family” “clinician” “healthcare provider” “health practitioner” “crisis intervention” “crisis care” “suicide prevention” “help-seeking” “suicide” “suicidal behaviour” “self-harm” “stigma” “discrimination” “attitude” “refuse service” “refuse treatment”

“anti stigma intervention” “stigma reduction intervention” “anti stigma program” “stigma reduction program” “anti stigma education” “anti stigma awareness”.

Example grey literature sources institutions/websites:

Australian Government - <https://www.nhmrc.gov.au/about-us/publications/clinical-practice-guideline-borderline-personality-disorder>

South Australian (SA) Mental Health Commission - <https://samentalhealthcommission.com.au/wp-content/uploads/ActionPlanPeopleLivingBPD.pdf>

Mental Welfare Commission for Scotland - <https://www.mwcscot.org.uk/sites/default/files/2019-06/nov2018bpd_report_final.pdf>
