# Supplementary material for: Structural stigma and its impact on healthcare for consumers with borderline personality disorder: protocol for a scoping review
Source: Syst Rev. 2021 Jan 11;10:23. doi: 10.1186/s13643-021-01580-1 (PMC7798332; doi:10.1186/s13643-021-01580-1)
Supplement: Supplementary file 3 — Additional file 3. Draft search strategy for PsycINFO database. [file 13643_2021_1580_MOESM3_ESM.docx]

**Additional file 3: Draft search strategy for PsycINFO database**

| **Search**  **No.** | **Searches** |
| --- | --- |
| 1 | Borderline Personality Disorder/ |
| 2 | Personality Disorders/ |
| 3 | (borderline adj4 (person* or client* or patient* or consumer* or carer* or famil*)).tw,id. |
| 4 | (BPD or BPDs).tw,id. |
| 5 | (personality adj4 disorder*).tw,id. |
| 6 | (emotional* unstable adj4 (person* or client* or patient* or PD or PDs or state* or disorder*)).tw,id. |
| 7 | or/1-6 |
| 8 | crisis intervention/ or suicide prevention/ or risk/ |
| 9 | crisis intervention services/ or emergency services/ or health services/ or mental health services/ or mental health care/ or primary health care/ |
| 10 | suicid*/ or self-destructive behav*/ or attempted suicide/ or suicidology/ or self-injurious behav*/ or suicidal ideation/ or self-mutilat*/ |
| 11 | (cris* or acute or emergenc* or critical).tw,id. |
| 12 | (help-seeking or help seeking behav*).tw,id. |
| 13 | (suicid* or self-harm or self-injur* or (self adj3 (harm or injur*))).tw,id. |
| 14 | or/8-13 |
| 15 | stereotyping/ or stigma*/ or prejudice/ or discrimination/ or marginali*/ or exp attitude/ |
| 16 | ((negative or positive) adj3 attitude*).tw,id. |
| 17 | (discriminat* or marginali* or reject* or exclu* or stigma*).tw,id. |
| 18 | (health services adj4 (experienc* or perspective* or perception* or view*)).tw,id. |
| 19 | (refus* or den*) adj3 (service* or treat*).tw,id. |
| 20 | ((anti-stigma or anti stigma or stigma-change or stigma reduction) adj3 (intervention* or program* or strategy* or education* or awareness)).tw,id. |
| 21 | or/15-20 |
| 22 | 7 and 14 and 21 |
| 1. 23 | limit 22 to english language |
