# Supplementary material for: Structural stigma and its impact on healthcare for consumers with borderline personality disorder: protocol for a scoping review
Source: Syst Rev. 2021 Jan 11;10:23. doi: 10.1186/s13643-021-01580-1 (PMC7798332; doi:10.1186/s13643-021-01580-1)
Supplement: Supplementary file 4 — Additional file 4. Draft data extraction tool of included studies. [file 13643_2021_1580_MOESM4_ESM.docx]

**Additional file 4: Draft data extraction tool of included studies**

| **Author,**  **Year, Country** | **MMAT Appraisal** | **Aims/**  **Purpose** | **Population Type/ Sample Size** | **Clinical Area/**  **Setting** | **Study Design/Data Collection** | **Activities/**  **Interventions** | **Main Findings** |
| --- | --- | --- | --- | --- | --- | --- | --- |
|  |  |  |  |  |  |  |  |
|  |  |  |  |  |  |  |  |
|  |  |  |  |  |  |  |  |
