# Supplementary material for: Structural stigma and its impact on healthcare for consumers with borderline personality disorder: protocol for a scoping review
Source: Syst Rev. 2021 Jan 11;10:23. doi: 10.1186/s13643-021-01580-1 (PMC7798332; doi:10.1186/s13643-021-01580-1)
Supplement: Supplementary file 5 — Additional file 5. Draft data extraction tool for structural issues associated with BPD-related stigma in healthcare systems. [file 13643_2021_1580_MOESM5_ESM.docx]

**Additional file 5: Draft data extraction tool for structural issues associated with BPD-related stigma in healthcare systems**

| **Author,**  **Year, Country** | **Population Type**  (BPD consumers, carers/ families, clinicians) | **Structural policies and procedures**  (including funding allocation) | **Clinical Practice**  (stigmatising norms, attitudes, behaviours) | **Main Findings** |
| --- | --- | --- | --- | --- |
|  |  |  |  |  |
|  |  |  |  |  |
|  |  |  |  |  |
